# Progress in Integrative Biomaterial Systems to Approach Three-Dimensional Cell Mechanotransduction

**DOI:** 10.3390/bioengineering4030072

**Published:** 2017-08-24

**Authors:** Ying Zhang, Kin Liao, Chuan Li, Alvin C.K. Lai, Ji-Jinn Foo, Vincent Chan

**Affiliations:** 1 Department of Chemical Engineering, Khalifa University, Abu Dhabi 127788, UAE; chemicalbio@gmail.com; 2 Department of Aerospace Engineering, Khalifa University, Abu Dhabi 127788, UAE; kin.liao@kustar.ac.ae; 3 Department of Biomedical Engineering, National Yang Ming University, Taipei 11221, Taiwan; cli10@ym.edu.tw; 4 Department of Architecture and Civil Engineering, City University of Hong Kong, Tat Chee Avenue, Kowloon, Hong Kong; alvinlai@cityu.edu.hk; 5 School of Engineering, Monash University Malaysia, Jalan Lagoon Selatan, 46150 Bandar Sunway, Selangor, Malaysia

**Keywords:** mechanotransduction, soft lithography, cell-matrix interactions, cell–cell interactions, cell traction force microscopy, 3D tissue mechanics

## Abstract

Mechanotransduction between cells and the extracellular matrix regulates major cellular functions in physiological and pathological situations. The effect of mechanical cues on biochemical signaling triggered by cell–matrix and cell–cell interactions on model biomimetic surfaces has been extensively investigated by a combination of fabrication, biophysical, and biological methods. To simulate the in vivo physiological microenvironment in vitro, three dimensional (3D) microstructures with tailored bio-functionality have been fabricated on substrates of various materials. However, less attention has been paid to the design of 3D biomaterial systems with geometric variances, such as the possession of precise micro-features and/or bio-sensing elements for probing the mechanical responses of cells to the external microenvironment. Such precisely engineered 3D model experimental platforms pave the way for studying the mechanotransduction of multicellular aggregates under controlled geometric and mechanical parameters. Concurrently with the progress in 3D biomaterial fabrication, cell traction force microscopy (CTFM) developed in the field of cell biophysics has emerged as a highly sensitive technique for probing the mechanical stresses exerted by cells onto the opposing deformable surface. In the current work, we first review the recent advances in the fabrication of 3D micropatterned biomaterials which enable the seamless integration with experimental cell mechanics in a controlled 3D microenvironment. Then, we discuss the role of collective cell–cell interactions in the mechanotransduction of engineered tissue equivalents determined by such integrative biomaterial systems under simulated physiological conditions.

## 1. Introduction

During tissue regeneration, the geometrical and mechanical cues of the surrounding microenvironment have been shown to regulate cellular responses, including migration, proliferation, differentiation, and apoptosis, etc. [1,2]. As such, tissue engineering traditionally refers to the development of various types of biomaterial scaffolds with specific bulk properties, such as porosity, microarchitecture, and compliance for extensive applications in cell therapy and tissue regeneration [3]. Although biomaterial scaffolding acts as a three-dimensional (3D) support for cell growth, it does not provide a highly engineered microenvironment with precise control in the location and morphology of various types of cells. Such spatial control is important for reestablishing the intricate organizations in the functional subunits of a typical organ. To overcome the limitations of biomaterial scaffolds, two-dimensional (2D) micropatterning of cells on various substrates has been exploited, with several techniques emerging, including microcontact printing [4], microfluidic patterning [5], photolithography [6,7], and plasma polymerization [8]. To date, surface features with spatial resolution of approximately 1 um can be fabricated by these techniques [9]. Increasingly, the 3D fabrication of precise microscale features which is not achievable with synthetic based approaches (e.g., hydrogel synthesis) is critical not only for controlling cell placement, but also for presenting spatially-controlled biological signals for the development of functional tissue constructs in vitro or in vivo [10]. In order to develop 3D micropatterned biomaterial scaffolds, several technical requirements in material selection, including mechanical properties, biocompatibility, and processability, must be thoroughly addressed for specific applications [11]. Recently, the advancement in 3D fabrication techniques has opened the possibility of attaining accurate spatial control of multiple cell types in engineered tissue equivalents. More importantly, such enabling technology facilitates the integration of cellular mechanical probes with a model microenvironment for studying intricate phenomena in mechanobiology [12]. Therefore, a timely review on the recent development of 3D cell patterning techniques in relation to the emerging investigations of 3D cellular mechanotransduction will highlight the importance of a generally ignored issue of mechanobiology for the design of tissue engineering products. 

## 2. Cell Mechanotransduction

**Figure 1:**
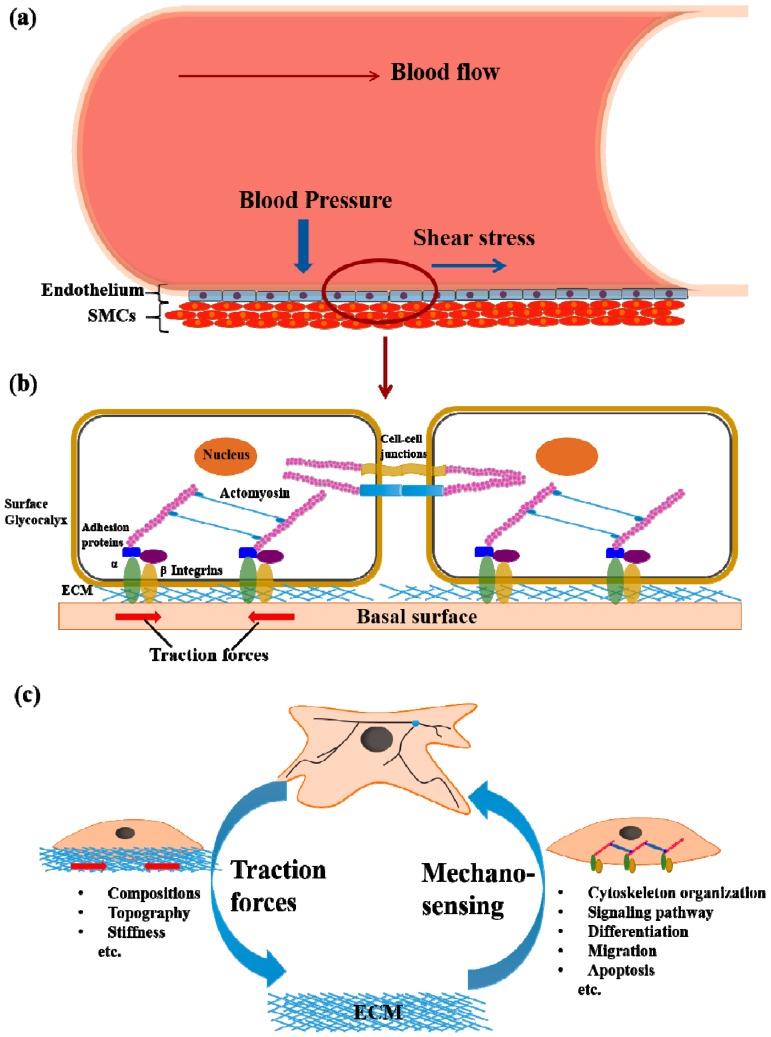
Schematic illustrations of (**a**) endothelial cells that form the interior surface of blood vessels subjected to stresses from the flowing blood; (**b**) The cell–cell junctions and cell–substrate tractions exerted by endothelial cells; (**c**) Positive feedback in cell–extracellular matrix (ECM) mechanotransduction. SMCs: smooth muscle cells.

Mechanotransduction, which generally occurs at the cell–extracellular matrix (ECM) interface and cell–cell contacts, is the transmission of mechanical forces to biochemical signals and vice versa for the regulation of cellular physiology. Mechanical force fields in the 2D or 3D space containing cells and ECM, either in the form of externally applied forces or cellular traction forces produced by the cytoskeleton, have been intensely studied due to their important roles in maintaining homeostasis in tissues in vivo. Although the involvement of cell traction force (CTF) on cellular signaling and physiological function has been revealed, the precise mechanism of mechanotransduction in 3D systems remains to be elucidated [13]. In the physiological microenvironment, both cells and subcellular organelles can sense mechanical stresses from various sources, such as shear stress of flowing blood, mechanical stress from the surrounding ECM, and contractile forces from adjacent cells [13]. There are significant differences between external forces and cell-generated forces, which can be characterized from the differences in magnitude, direction, and distribution. However, certain indications on the existence of tight coupling between external applied forces and cell-generated forces have been highlighted [14,15]. For instance, biomacromolecules, such as carbohydrate-rich glycocalyx, which are found on the apical surface of vascular endothelial cells, have been shown to transmit fluid shear stress under blood flow to the cortical cytoskeleton [16]. In the mechanotransduction of the cardiovascular system, shear stress induced by flowing blood has been known to deform the endothelial cells at the inner wall of blood vessels and to trigger a cascade of cell signaling for the regulation of vascular physiology (Figure 1a). The endothelium mechanobiology, which leads to the generation of CTF (red arrows on Figure 1b indicate the direction of contractile forces), is actually governed by the highly synchronized interactions between external mechanical forces, cell–ECM adhesion, cytoskeletal protein binding, blood vessel stretching, cell–cell junction formation, and basal membrane mechanics, etc. (Figure 1b). Therefore, the mechanotransduction of cell layers will be thoroughly discussed herein by focusing on the feedback mechanisms of cell signaling with adjacent cells or ECM.

In the first step of cell–ECM interaction, the binding between integrin and ECM protein triggers the assembly of focal adhesions (FAs) which in turn link the integrins to the actin filaments of the cytoskeleton with the help of adaptor proteins (e.g., talin, alpha actinin, vinculin) [17]. Moreover, FAs have been known to stabilize the adhesion sites by synergistic interactions of numerous signaling proteins (e.g., FAK, Ras, src) [18]. The biochemical processes involved in the generation of contractile force in cells after adhesion and spreading on the ECM or substrate are triggered by actomyosin interactions and actin polymerization [19,20]. These cell-generated forces, as mentioned above, are commonly known as CTF which are transmitted forward to the ECM through integrin, and then feed back to biochemical signals from ECM proteins to cytoskeletal proteins [21]. A series of molecular biology studies have revealed that CTF is associated with major cellular functions through their involvement in various signaling pathways. In smooth muscle cells (SMCs), CTF generation is mainly regulated by the Rho-kinase/ROCK pathway, which directly affects mitogen-induced DNA synthesis [22] and FA assembly [23]. As such, the presence of CTF plays a key role in modulating cell differentiation, migration, and apoptosis, and in maintaining cell homeostasis in the local microenvironment [24]. An emerging question on the intricate mechanisms of CTF regulations leading to various cellular responses needs to be better addressed. For instance, it has been demonstrated that the mechanotransduction response of cells towards principal substrate characteristics, such as stiffness and nanotopography, is linked to the talin-mediated mechanical regulation of the molecular clutch, integrin clustering, and FA dynamics [25,26].

Most anchorage-dependent cells, except endothelial cells and epithelial cells, are functional only in a 3D microenvironment in vivo. Thus, artificial 3D ECM networks have been developed as model systems for cell culture and bio-functional studies. In general, collagen in the form of a 3D network or a 2D gel has been widely used in various semi-quantitative assays of cell adhesion, biological functions, and chemotaxis. For instance, the phosphorylation level of focal adhesion kinase (FAK) in fibroblasts, which is associated with cell spreading, is reduced in 3D collagen gel matrix compared to that on 2D collagen gel, while the formation of FAs of fibroblasts in the 3D collagen gel matrix are triggered by the clustering of α_5_β_1_ integrins instead of α_v_β_3_ integrins found on the 2D collagen gel [27]. Comparing 3D to 2D collagen cultures of SMCs, an upregulation of p21 and transforming growth factor beta 1 (TGFβ1) expression, an upregulation of extracellular signal–regulated kinases (ERK) phosphorylation, and a downregulation of FAK phosphorylation have been found, which supports the role of geometrical factors of the microenvironment on cell proliferation and FA formation [28,29]. Similarly, an increase of matrix stiffness in 3D collagen network has been shown to promote the invasive phenotypes of mammalian epithelial cells, such as Rho-mediated contractility [30]. Also, primary dermal fibroblasts have switched from lamellipodia-based migration to lobopodia-based migration in response to the shift of the mechanics of 3D gel matrix from nonlinear elasticity to linear elasticity [31]. All results as mentioned above strongly suggest that the physical and mechanical properties of the ECM or biomaterial directly moderate the intricate mechanisms of cell adhesion and migration. Such influences have elicited the interplay between the mechanotransduction of adherent cells and molecular architecture of the biomaterial scaffold or ECM network.

The mechanotransduction of a single cell on 2D biomaterial has been extensively studied during the past two decades. CTF generated by the cytoskeletal remodeling of adherent cells is exerted to the ECM or material interfaces of the surrounding microenvironment through the anchoring of FAs. At the same time, the CTF of individual cells are influenced by both the surrounding ECM and neighboring cells. Under in vivo microenvironment, a cell migrates with the CTF generation on its surrounding ECM by synchronizing the protrusion and contraction at its leading edge and trailing edge, respectively. On the other hand, the collective mechanotransduction of multicellular aggregates in a 3D biomaterial, which has been studied to a lesser extent, likely influences major cellular physiology, including morphogenesis, wound healing, and embryogenesis. A recent study has reported the successful measurement of a CTF map exerted by an advancing epithelial cell sheet on a hydrogel matrix [32]. Moreover, CTF in relatively small cell colonies composed of 1–27 cells on 2D silicone gel has been found to be localized at the periphery of cell aggregate and is positively correlated with the colony size [33]. Interestingly, another group has demonstrated that CTF is generated from cells locating further behind the front edge of an advancing cell sheet instead of cells at the migrating front boundary during collective migration [34]. In comparison to the mechanotransduction of single cells, the maximal traction forces and stresses near the edge of the cell monolayer are significantly higher [35]. Furthermore, the higher responsiveness of a cell sheet towards a stiffness gradient on a planar substrate in comparison with a single cell suggests that cell–cell interaction enhances the collective cell migration of a cell sheet [36]. Recently, a key endocytic protein known as RAB5A has been shown to trigger collective migration of a cell population in originally stagnant epithelial monolayers [37]. 

In general, the mechanical properties of native tissues vary significantly according to the functional requirements of specific tissues or organs, and act as indicative parameters of the progression in certain disease, e.g., muscular dystrophy [38]. As the mechanotransduction of cells is related to cytoskeleton remodeling, ECM mechanics, and ECM protein composition, extensive studies have been focused on the fabrication of substrates with variable stiffness, adhesive ligands, and micropatterns in order to elicit the physiochemical factors for governing cell mechanics and function [39]. Cells exert CTF on the surrounding microenvironment, and at the same time moderate their cellular responses towards the mechanical feedback signals obtained from the surrounding ECM (Figure 1c). In detail, the interaction between individual cells and the ECM through interconnected pathways of receptor-mediated signal transductions, CTF generation, and external matrix mechanics dictate the expression of specific genes and phenotypes. At the same time, the elucidation of collective mechanics in cohesive cell layers as well as its feedforward-feedback responses with surrounding microenvironments are required for determining the working principle in tissue morphogenesis and regeneration. With the help of CTF measurements, the cell signaling pathways involved in many physiological functions and pathological processes of single cells have been revealed in more detail [15]. In addition to the mechanotransduction of individual cells, CTF measurement of cell populations will make the in vitro biophysical study of morphogenesis possible. It is believed that a thorough understanding of the intricate interplay between cell mechanics and cell–ECM interactions will facilitate the development of biofunctional and mechanoresponsive biomaterials for tissue regeneration and clinical diagnostics.

Mechanical stresses from the physiological microenvironment, including substrate, flowing fluid, ECM, and neighboring cells, directly influence the collective responses of cell layers, e.g., the regulation of the vasoactivity of blood vessels. A typical mechanosensing event in epithelial and endothelial tissues is triggered by the formation of cell–cell junctions, which contain adherens junctions, gap junctions, and tight junctions. For instance, adherens junction formation has been shown to induce actin polymerization in the cytoskeleton of adjacent cells through the interactions of various signaling proteins around the cytoplasmic domains of adherens junctions [40,41,42]. Moreover, cell–cell interaction has led to the inhibition of cell proliferation and migration which is known as “contact inhibition”. In the 2D culture of epithelial cells, cells which are sparsely distributed on the substrate proliferate well, while the growth of a densely packed cell monolayer is impaired by the formation of cell–cell contacts [43,44]. The phenomenon of contact inhibition of cell proliferation in epithelial cells also exists in different types of cells [45,46]. Quantitative characterizations of contact inhibition dynamics in confluent cell monolayers with different cell densities have shown that the interfacial contact formation between adjacent cells is necessary but not sufficient for causing growth inhibition [47]. It has also been suggested that mechanical compression may provide an inhibitory signal for cell division [48]. 

In addition to the inhibition of cell proliferation, cell-cell junction formation between neighboring cells provides a cooperative effect to transmit appreciable normal stress, which guides the direction of cell monolayer migration along the course of minimal intercellular shear stress [49]. As epithelial cells require stable cell–cell adhesions and mesenchymal cells rely on transient and dynamic cell–cell contacts, the strategies of collective cell migration in various cell types are different based on their particular physiological functions [50]. A recent study has shown that the chase-run phenomenon between placode cells and neural crest cells was involved in both chemotaxis and *N-*cadherin signaling transduction, which in turn led to coordinated migration of different types of tissues [51]. With the hypothesized phenomena of mechanical coupling between neighboring cells, cell–cell interactions can be sufficient to guide the direction of collective cell migration, without the presence of a physical cue [52,53,54]. On the other hand, understanding the principles and mechanisms involved in collective cell migration remains a tremendous challenge, because the mechanical stresses of cell layers are difficult to probe by conventional experimental techniques. CTF, which is a known mechanical property, has been adopted as a model biophysical parameter for analyzing the intricate mechanotransduction of cell monolayers [55,56,57].

## 3. 3D Fabrications of Polymeric Biomaterials

In general, the most commonly used synthetic polymers for 3D fabrication of biomaterial scaffold include polyglycolic acid (PGA) [58], poly-caprolactone (PCL) [59], polydimethylsiloxane (PDMS) [60], and polyethylene glycol (PEG)-based hydrogels [61]. For example, PGA and its copolymers are widely used in the fabrication of various biomaterial scaffolds for tissue engineering applications because they are biodegradable and non-toxic [62]. However, the native surface of PGA does not provide the biological cues for direct cell attachment and regeneration. Thus, the modification of PGA with biologically active ligands is essential for cell culture applications, e.g., the addition of β-tricalcium phosphate (β-TCP) in porous 3D PGA scaffolds has been required for treating bone defects [63]. Moreover, polylactic-co-glycolic acid (PLG) copolymers have been used to design biomaterial scaffolds incorporated with controlled release capability of essential biomacromolecules, such as vascular endothelial growth factor, in vivo [62]. The PLG system mentioned above has facilitated blood vessel development and enhanced local vascularization during tissue regeneration [64]. Besides, microporous membrane composed of poly-d, ᴌ-lactide-co-glycolide (PLGA) has been applied for the 3D stacked culture of hepatic tissues [65].

**Figure 2:**
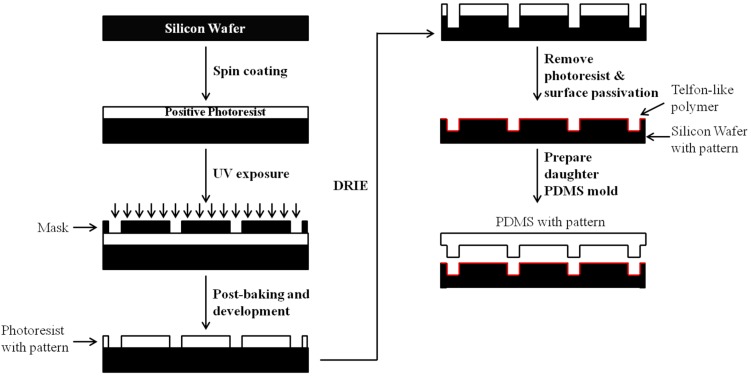
Schematic illustration on the fabrication process of a micropatterned polydimethylsiloxane (PDMS) scaffold by combining lithography and deep reactive ion etching (DRIE).

In spite of the intense development of microporous polymeric scaffolds, there has been a lack of 3D biomaterial systems with precise microscale features for simultaneous cell culture and biomechanical measurement. As such, Shen et al. have developed a high-aspect ratio microchannel by UV embossing of an UV polymerizable biodegradable macromer in a liquid formulation composed of poly-e-caprolactone-r-ᴌ-lactide-r-glycolide diacrylate [66]. In detail, the master silicon (Si) mold is prepared from a lithography system which uniquely combines deep reactive ion etching (DRIE) with passivation treatments (Figure 2). After obtaining the daughter PDMS mold, an UV resin liquid formulation and a polyester film are successively loaded onto the PDMS mold. Lastly, the resin is polymerized under UV illumination and separated from the PDMS mold and polyester film [67,68]. The depth of the micropatterned biodegradable scaffold synthesized by this method can reach up to 70 μm. Furthermore, the layer-by-layer (LBL) process has been successfully applied to build multilayers of confluent SMCs on the micropatterned biodegradable scaffold as mentioned above, and to trigger the expression of the contractile phenotype of SMCs [69]. In addition to the formation of cell patterns, effective mass transfer of dissolved gas and essential nutrients to cells bound on the biodegradable scaffold are critical for maintaining cell viability and functionality. To address the limitation of mass transfer, Sarkar et al. [59] have developed a porous micropatterned PCL by combining soft lithography, melt molding, and PLGA micro/nanoparticles leaching. The group has further demonstrated that the diffusion rate of culture media into the PLGA-leached PCL scaffolds mentioned above was enhanced by six times in comparison with that through the non-porous PCL scaffolds. Later on, a biodegradable microstructured polycaprolactone construct functionalized with an adhesive layer of polyethylene glycol-diacrylate (PEG-DA) gel was incorporated into a 3D composite structure with a layer of vascular smooth cells with precisely controlled cell orientation and geometry [70]. Until now, most emerging techniques, as mentioned above, mainly focus on the precise 3D fabrication of microscale patterns in biomaterial scaffolds for facilitating the formation of tissue equivalents, instead of probing intricate cellular behavior in a controlled 3D microenvironment. 

Interestingly, Deutsch et al. [60] have developed a new fabrication process for microgrooved topographical cues on a PDMS scaffold by combining photolithography and soft lithography. Briefly, the silicon wafer is spin-coated with a photoresist layer and exposed to UV light through a contact mask. The micropatterned PDMS scaffolds are created by casting and curing of siloxane oligomers and siloxane cross-linkers on the wafer surface. The fibronectin-coated PDMS scaffolds fabricated by this method have been shown to direct the spatial organization of cells and extracellular matrix [71]. Based on its ideal biocompatibility, polyacrylamide hydrogel has been used as a model system for studying the biophysics of cell migration, e.g., chemotaxis [34]. In another study, PEG-based hydrogel microstructures were fabricated on glass substrates by using photolithography-based patterning [61,72]. In detail, a glass substrate was first functionalized with 3-(trichlorosilyl)propyl methacrylate, then spin-coated with a PEG derivative and a photoinitiator, and finally exposed to UV light through a photomask for PEG crosslinking. Moreover, the PEG-based hydrogel was successfully fabricated into a cylindrical or cubic shape for the encapsulation of cells, detection of drug–drug interaction, and formation of tumor spheres [73,74,75]. Hahn et al. [76] have further developed confocal-based laser scanning lithography for 3D surface patterning (feature size ~5 μm) of PEG-based hydrogel substrates. The micropatterned PEG-based hydrogels embedded within independently fabricated PDMS housings are applied as a microfluidic device to study cell viability and metabolic activity, which contributes to the emerging applications for in vitro diagnostics and regenerative medicine [77]. At the same time, the PEG-based hydrogels can be further developed into a porous 3D structure to control spatial organization and enhance cell binding affinity for tissue morphogenesis and angiogenesis promotion [78,79]. In addition to lithographic patterning, other bottom-up approaches, such as electrospinning, nanoimprinting, anodization, and phase separation, have been applied to fabricate 3D patterns on biomaterials [80]. 

Naturally-derived polymers, such as proteins and polysaccharides, are ideal materials for the fabrication of biomimetic 3D scaffolds with superior biocompatibility compared to synthetic polymers. Collagen is one of the most important natural polymers widely found in most extracellular matrix (ECM) surrounding tissues in vivo as it serves as an adhesive ligand for major cell types [81]. Thus, phase change ink-jet printing and indirect 3D printing techniques have been applied to fabricate collagen scaffolds with micropatterns, such as internal channels and capillary networks [82,83]. For instance, indirect 3D printing of collagen involves three steps. Firstly, a negative mold is created by a solid freeform fabrication technique which produces freeform solid objects directly from computer-aided design without part-specific tooling or human intervention [84,85]. Secondly, a collagen solution is casted into the mold and solidified at low temperature. Lastly, the patterned collagen scaffold is recovered by the dissolution of the mold with ethanol, followed by critical point drying with liquid carbon dioxide. Another preferred method for fabricating collagen-based scaffolds with uniform pore structure is homogeneous freeze drying under a controlled freezing rate [86]. Cell attachment and viability on the freeze-dried 3D collagen scaffold are shown to be influenced by specific surface area and pore size of the resulted scaffold [87]. 

Besides protein, chitosan, which is formed from the deacetylation of chitin, is an important naturally-derived polysaccharide for fabricating biomaterial scaffolds in various applications of tissue engineering, such as hepatocyte regeneration [88,89]. In a pioneering study, a rapid prototyping robotic dispensing (RPBOD) system has been developed to fabricate 3D scaffolds of chitosan and chitosan-hydroxyapatite (HA) with reproducible macropore architecture by injecting chitosan and chitosan–HA solution (in acetic acid) into a mixture of sodium hydroxide solution and pure ethanol (in ratio of 7:3) [90]. Alternatively, a hydrogel with molecularly engineered filaments has been formed from the self-assembly of oligopeptides to mimic the ordered microporous structure of native ECM for 3D cell culture [91,92]. Recently, one self-assembly oligopeptide (SAP) designed with specific motifs for enhancing cell adhesion, cell differentiation, and bone marrow homing has been successfully developed into nanofibers for the functional maintenance of mouse adult neural stem cells in a 3D microenvironment. Also, SAP gels with high peptide concentration have been generated from a standard solid phase synthesis method for hosting cells inside their matrices. To date, the SAP gels have been further engineered with tunable physical properties such as nano-scale architecture and biological functionality for various applications in tissue engineering, such as vascular graft [93,94]. In spite of the recent advances in the 3D fabrication of natural materials, the quantitative correlation between scaffold properties and cellular responses for effective tissue regeneration remains to be elucidated. In general, a 3D biomaterial scaffold should mimic the physical microenvironment and biochemical cues of native ECM as closely as possible. Therefore, a better understanding of cell–biomaterial interaction and bidirectional mechanotransduction within the 3D microenvironment will simultaneously aid the design of superior biomaterial scaffolds for tissue regeneration and the development of a model experimental platform for the study of cell mechanotransduction. 

## 4. Recent Progress in Cellular Biomechanics

It is now known that the bulk elasticity (ranging from rigid to soft material) and surface topography of a biomaterial directly modulates the contact mechanics of cells, while microfabrication technologies, such as soft lithography, provide the spatial control of cell populations on the biomaterial scaffold. Various types of cells, such as fibroblasts, SMCs, and hepatocytes, respond differently to substrates with a range of elasticity through intricate interplay between ligand–receptor binding, mechanotransduction, and cytoskeleton remodeling [95]. For instance, cell spreading and locomotion speed of normal rat kidney epithelial cells has been demonstrated to reduce and increase, respectively, on softer polyacrylamide gel substrates [96]. Moreover, 3T3 fibroblasts have demonstrated preferential migration, known as durotaxis, over an elasticity gradient from a soft region to a stiff region on a polyacrylamide gel substrate, but not vice versa on the same substrate [97]. Interestingly, a recent discovery has proven that the migration of multiple cell aggregates is responsive to certain perturbations of cell–cell adhesion, such as the downregulation of adherens junctions [98]. Recently, topographical cues such as micropillars [99] and nanoparticulates [100] have also been shown to effectively influence the contact mechanics and biological functions of adherent cells. 

In general, the communication between cells and the apposing ECM or biomaterial scaffold leading to cell growth and motility is driven by intricate mechanotransduction through the active reorganization of the cytoskeletal network in the cytoplasm [101,102]. First of all, cell migration speed has been demonstrated to follow a biphasic dependence on the density of immobilized adhesive ligands, as predicted by a seminal mathematical model [103], and later on validated in experimental measurement [104]. At the same time, the increase of ECM stiffness has been shown to enhance cell motility as cells exert higher contractile forces towards stiffer substrate during cell body attachment, detachment, and displacement [105]. The geometry of the 2D biomaterial sub-region functionalized with adhesive islands can be precisely engineered with the use of microcontact printing techniques which are instrumental for studying the combined effects of geometrical constraint and biological recognition on cellular physiology. Several studies have shown that cell adhesion is not only triggered by the binding between membrane-bound integrin and adhesive ligands, but also related to the subsequent events, such as biochemical signal transduction and mechanical deformation of intracellular organelles in the adherent cells [106]. It has been revealed that both the formation of FAs and the emergence of actin stress fibers of adherent cells are dependent on ECM stiffness. Interestingly, the optimal value of substrate stiffness for supporting maximal migration is correlated with the concentration of ECM ligands covalently attached to the substrate [104]. Recently, a group has even demonstrated that nanotopographical features deposited on a planar glass substrate are sufficient to promote the maturation of neural networks [107].

Classical model systems for studying the mechanotransduction of cells at the cell–substrate interfaces have been made possible with the development of experimental biophysics since 1980. In the first report, the elastic distortion and wrinkling of a silicone substrate induced by the adherent cells through cytoskeleton remodeling during cell locomotion have been observed by optical microscopy [108], and can be further quantified through the variation of substrate elasticity [109,110]. Thereafter, Balaban et al. have combined the transparent micropatterned elastomer and FA characterization through the expression of green-fluorescent protein (GFP)-labeled vinculin in adherent cells for elucidating the relationship between the assembly dynamics of FAs and the alteration of CTF [111]. The method mentioned above mainly hinges on the use of a non-wrinkling substrate with micropatterns composed of dots or lines for the determination of cell-induced deformation of the material’s surface. 

In a seminal study, Tan et al. developed a microfabricated array of elastomeric microposts, each independently acting as a deformable structure to probe the mechanical transduction between the adherent cells and apposing substrates [112]. With the use of a micropost as the mechanical probe, the map of highly localized deformation caused by the CTF across the entire substrate can be determined from beam theory [113]. By changing the height of the micropost, a series of micropost arrays reported with a range of substrate rigidity further demonstrates the influence of microscale geometry and elasticity on cell morphology, FA organization, and global cell contractility [114]. By combining micropost array technology with ultrahigh-resolution cell imaging, the precision map of CTF with a sensitivity of 500 pN and the nanoscale resolution of individual force-bearing FAs have been simultaneously measured [115]. Moreover, the micropost array has been developed to probe the real-time mechanotransduction of entire cells through the integration with a cell stretching device, which provides a way of tuning the external force exerted on live cells [116,117]. Since microposts have been commonly fabricated on elastomeric polymers such as PDMS, the functionalization of the PDMS surface with ECM proteins is essential to create a more physiological relevant microenvironment for cell adhesion. In addition to PDMS, polymeric hydrogels have been used in the fabrication of micropost arrays [118]. In detail, the hydrogel monomers, such as hydroxyethyl methacrylate, were mixed with a photoinitiator, followed by replica molding and UV exposure in order to partially polymerize the precursors in solutions. Secondly, photo-crosslinking of the solutions, as mentioned above, with ethylene glycol dimethacrylate leads to the formation of a stabilized hydrogel micropost array. Moreover, the stability and surface clustering of a range of high-aspect-ratio hydrogel microarrays under capillary forces have been studied by controlling the geometry and elastic moduli of individual microposts [119].

**Figure 3:**
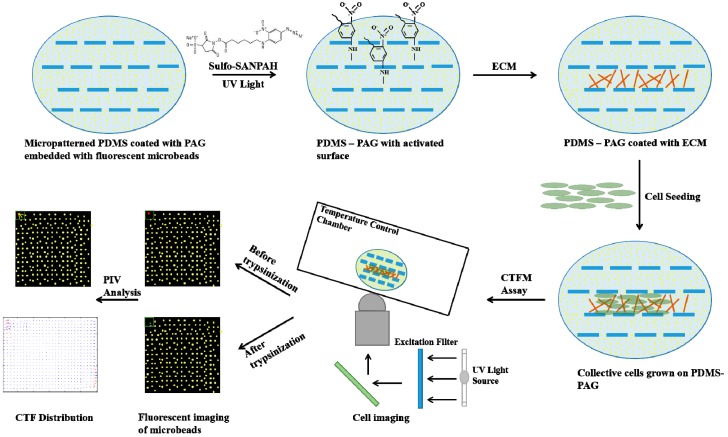
The schematic illustration for the making of PDMS microchannels with a polyacrylamide gel (PAG) coating for cell traction force microscopy measurement (Top views). The micropatterned PDMS is coated with PAG embedded with a thin layer of fluorescent microbeads. A UV-activated heterobifunctional cross linker, sulfo-SANPAH, is applied for ECM coupling. Cells are then seeded onto the activated surface for further study with cell traction force microscopy. After obtaining a pair of fluorescent images of the same frame before and after trypsinization, the deformation of the elastic substrate is determined and used for the CTF computation.

Another popular substratum for studying cell–substrate mechanotransduction is polyacrylamide-based hydrogel (Figure 3), a microporous material which has been proven to be an ideal experimental system for various cell types due to its tunable chemical and mechanical properties [120,121]. Firstly, polyacrylamide-based hydrogel can be easily tuned from an extremely soft to stiff material with a distinct elastic modulus by adjusting the concentration ratio of acrylamide and bis-acrylamide before the initiation of polymerization. Secondly, the polyacrylamide-based hydrogel can be loaded with fluorescently labeled microbeads which serve as positional markers for the detection of local deformation in selected regions of interest [122]. More importantly, polyacrylamide-based hydrogel can be linearly deformed in response to the applied biological forces and can be fully recovered to its original state upon the removal of force. Thirdly, the low thickness and transparent nature of polyacrylamide-based hydrogel ensures the detection of small displacements of the fluorescent beads with conventional fluorescence microscopy. Furthermore, the polyacrylamide-based gels are naturally non-adhesive for mammalian cells unless specific ECM molecules (e.g., type I collagen) are covalently coupled onto the surface. 

In addition to the micropost array and polymeric hydrogel, the microelectromechanical systems (MEMS) with on-line force transducers have determined the contractile force of adherent cells from the measured deflection and the spring constant in the enclosed microbeams [123]. In one typical MEMS design, the cell contraction force was measured from the deflections of micron-sized pads connected to a cantilever [124]. A substrate composed of an array of closely packed cantilevers has been designed with different tip geometry and spatial distribution for probing the local mechanical forces at the cell adhesion contact [125]. At the same time, the control of the geometric pattern of a cell population on planar MEMS can be achieved by using microcontact printing, as mentioned earlier. 

It is known that cell spreading and migration within 3D microenvironment is highly dependent on the topological properties [126] and mechanical stiffness [127,128] of the surrounding ECM or biomaterial scaffold. The stiffness of a 3D collagen gel matrix has been engineered with different degrees of crosslinking under the same collagen concentration in solution for probing the effect of gel matrix stiffness on cellular behavior. The result indicates that the increase of gel matrix stiffness enhanced the spreading of endothelial cells [129]. When cells are partially or completely embedded within a 3D matrix, the cellular responses are quite different from those observed in a 2D culture dish, e.g., FAs become smaller, more diffusively distributed, and changed in FAK composition in the cell cytoplasm [130,131]. Moreover, FA-associated proteins regulate the migration speed of live cells embedded in a 3D matrix through their distinctive roles in driving protrusion activity and matrix deformation, which are unimportant in 2D cell migration [132,133]. In contrast to individual cells, the spatial organization of FAs within multicellular aggregates cultured inside a 3D matrix remains to be fully elucidated. 

## 5. Cell Traction Force Measurement

Mechanotransduction is an emerging research area which requires the knowledge from several disciplines. A variety of methods have been developed to measure the CTF of both individual cells and collective cell populations during the past few years [118], including cell-populated collagen gel (CPCG) [119], thin silicone membrane [108], force sensor array [123], and improved micropost force sensor [112]. The reported force and spatial resolution of major force sensing techniques mentioned above have been summarized in Table 1 [134]. Currently, the most reliable and comprehensive method primarily developed for CTF measurement is the cell traction force microscopy (CTFM) assay, which was developed by Dembo and Wang in 1999 [135]. 

In CTFM, the fluorescent microbeads embedded in the substrate serve as positional markers to record and track the deformation caused by CTFs (Figure 3). By taking a pair of ‘force loaded’ and ‘null force’ images in the same region of interest, the deformation of the elastic substrate is determined and used for the CTF computation. By dividing images into overlapped windows with a constant distance, a pair of small windows respectively from ‘force loaded’ and ‘null force’ images are obtained and applied to calculate the displacements. There are two other versions of CTFM techniques that have been developed by Butler et al. [19] and Yang et al. [136]. Both methods rely on the setting window, and conduct the correlation computation by using Fourier transform. On the other hand, the bead displacement calculation used in the Dembo and Wang [135] method is not fixed to the paired windows as mentioned above. Butler et al. have developed explicit formulas for transforming the traction and displacement fields of microbeads to cell mechanotransduction parameters, such as the contraction moments and strain energy at cell-substrate interfaces [19]. While other approaches based on pixel windows ignore the local rotational displacements of microbeads, the method developed by Yang et al. incorporates a pattern recognition technique to track microbead movements for estimating the displacement field of the elastic substrate [136]. Most importantly, all CTFM associated methods need to overcome the challenge of the accurate measurement of the small displacements of fluorescent microbeads. In addition, all methods of data analysis mentioned above are limited to the positional mapping on a 2D substrate. The CTFM method can achieve a broader application if the CTF characterizations can be extended to 3D matrices. 

Recently, more efforts have been devoted to the development of novel experimental and numerical methods for probing 3D CTFs, which have similar elastic moduli and physiological features to in vivo situations. By combining laser scanning confocal microscopy with digital volume (3D) correlation, 3D full field traction can be computed by the cross-correlation function and displacement-gradient technique [126,137,138,139]. In this method, the 3D CTFs of single cells are mapped over the 2D surface between cell and polyacrylamide gel, and the dynamic CTFs during cell migration and locomotion are calculated. Similarly, Hur et al. have developed 3D CTFM techniques to probe the cell–matrix interaction of live bovine aortic endothelial cells (BAECs) on a polyacrylamide deformable substrate in real time [140]. Moreover, Delanoë-Ayari et al. have presented the temporal map of CTFs during the crawling of Dictyostelium cells on a soft hydrogel surface [141]. The differences among the two methods mentioned above are the maximum limit of bead displacement in the computation algorithms [142]. In comparison to the traditional linear deformation framework used in traditional CTFM, a large deformation formulation for characterizing the cellular displacement field in conjunction with the high resolution digital volume correction technique has been recently developed [143].

For 3D CTF measurements, i.e., for cells completely immersed within ECM, collagen rather than synthetic hydrogel is used as a model system. The challenges of using a naturally-derived 3D scaffold hinge on the difficulty in the control of its mechanical properties and in the limitation of its fabrication process [93]. Legant et al. have successfully encapsulated GFP (EGFP)-expressing cells in well-defined PEG hydrogel matrices, tracked the displacement of embedded fluorescent beads, and calculated the CTFs exerted by the entrapped live cells [144]. They have applied linear elastic theory and the finite element method to analyze the bead displacement map generated from confocal microscopy of hydrogels. Their results indicate that the stiffer the hydrogels is, the stronger CTF the cells exert on the surrounding 3D ECM. Strong inward forces have been revealed to locate predominantly near the long extensions of the encapsulated cell, while the shear tractions are the main type of CTF produced by the cells encapsulated in 3D hydrogel [144]. By applying a 3D type I collagen network for cell encapsulation, Gjorevski et al. [145] and Koch et al. [146] have obtained the 3D CTF mapping of single cells throughout the surrounding hydrogel matrix. One challenge of this method is the interpretation of the traction field from the cell types, like fibroblasts and certain tumor cells which can degrade surrounding collagen matrix and subsequently change the mechanical properties of the surrounding hydrogel during the CTFM assay [131]. 

Various external mechanical stimulations acting on cells from the surrounding microenvironment take the form of shape, topology, and rigidity, which trigger the cycles of mechanosensing, mechanotransduction, and mechanoresponse [147]. The exact mechanisms of mechanochemical signaling from extracellular mechanical forces to intracellular molecular recognitions remain unclear. To date, fluorescence resonance energy transfer (FRET)-based single-molecule spectroscopy of mechanosensory molecular beacons has been validated with the high sensitivity required for probing forces in the piconewton scale [148]. Such a technique opens up the new possibility for tracking the propagation of molecular forces within cells during mechanotransduction. By incorporating FRET into CTFM, valuable information connecting the cascade of mechanical stimuli propagations to the identification of mechanosensitive proteins can be gathered conveniently. One of the possible exploitations of the study of cell mechanics is to elucidate the developmental processes of mesenchymal stem cells in response to external matrices of different underlying rigidities. For instance, the differentiation into various cell lineages ranging from neurogenic cells on a softer matrix, myogenic cells that resemble muscle tissues on a stiffer matrix, to the osteogenic cells on the most rigid matrix, are identified on various crosslinked-collagen matrices [100].

**Figure 4:**
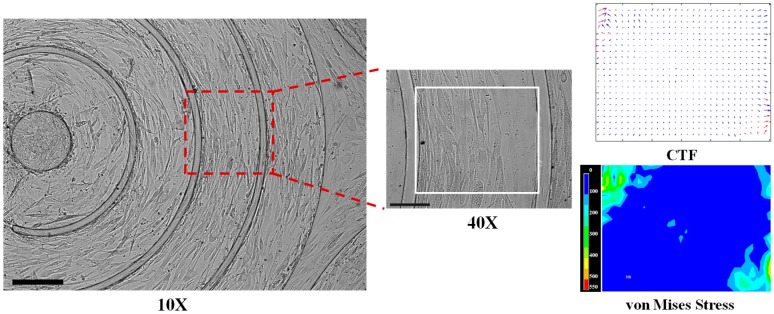
The distributions of CTFs and von Mises stresses of a confluent SMC sheet aligned in the circumferential direction. Scale bars are 250 µm and 100 µm in the 10X and 40X images, respectively.

By combining soft lithography with CTFM techniques, recent work focusing on confluent cell monolayers cultured on a micropatterned polymeric substrate has been carried out to elucidate the cell–cell interactions and intracellular mechanics, such as the collective CTF distribution of SMC layers during the emergence of contractile phenotypes under a controlled 3D microenvironment (Figure 4) [149]. In the vascular physiology of the endothelium, the effect of confluency of an endothelial cell monolayer on the tensions at adherens junctions and the cytoplasm under both static and shear flow conditions has been revealed [150]. A few groups have also shown that the spatial distribution of CTFs exerted by cohesive cell colonies is significantly concentrated at the periphery of the colony [33,151]. A similar homogeneous zone of lower CTF or von Mises stress was observed in the center of a circumferentially aligned SMC sheet, as shown in our recent study (Figure 4). In other words, collective CTF is strongly influenced by the shape of the adhesive zone prescribed on the micropatterned substrate. For better understanding of collective cell activities, novel CTFM has been developed to show the dynamic traction domains and the compressions among cell clusters [152]. To quantify the cell-generated mechanical stress in situ within living tissues, an emerging technique based on 3D functionalized fluorocarbon microdroplets has been developed for studying the mechanical properties of aggregates of mammary epithelial cells [153]. Moreover, the shape deformation of the microdroplets (with similar size to individual cells) which are microinjected into the tissue of mouse embryo can be used to calculate the anisotropic stresses generated by epithelial cell colonies via fluorescent imaging and computerized analysis.

Although 3D CTFM has attracted much attention, the techniques are still immature and not applicable to various experimental systems. Several challenges, including the fabrication and understanding of the 3D matrix, the influences of nanotopography, the techniques of high resolution 3D imaging, and the necessity for complex computational algorithms, need to be overcome for promoting the general adaptations of this potentially powerful technique [154]. In addition, various methods for probing the collective cell traction field are affected by the intricate mechano-sensing mechanisms of cells within a 3D microenvironment. Considering that multiple-cell sheets instead of single cells participate in most physiological processes, there is an essential need to develop measurement methods based on biomaterial innovations for 3D CTF that result from cell populations.

## 6. Conclusions

In order to study cell/tissue mechanotransduction under physiological conditions, a transition from 2D to 3D biomaterial systems with prescribed microscale features is necessary. 3D micropatterning tools for biomaterial fabrication can improve our knowledge about the influence of microenvironments, like the composition, topography, stiffness, etc., on cellular functions, physiological regulations, and pathophysiological progressions. While the general principles of the design for an appropriate 3D scaffold have been formulated, the better understanding of the mechanosensory responses of cells within 3D microenvironments will in turn facilitate better scaffold design. Since cells undergo complex mechanotransduction when they attach to and spread on a substrate surface, new advances in the development of integrative biomaterial systems for probing the CTFs of single cells as well as cell layers have been recently achieved. Given CTF as an ideal model to analyze the complex mechanotransduction of multiple cells, 3D CTFM and derived techniques for the measurement of collective CTF will become increasingly important. 

## Figures and Tables

**Table 1:** Force Sensitivity of Cellular Forces Measured with Selective Cell Mechanics Techniques

TECHNIQUE	FORCE SENSITIVITY
Optical Tweezers	1–100 pN
Atomic Force Microscope	10–10^5^ pN
Magnetic Tweezers	10–10^3^ pN
Gel Wrinkling Method	10–100 nN
Micropost Deformation	1–100 nN
Cell Traction Force Microscope	10–10^6^ pN
